# Sex and Gender Transition of People With Multiple Sclerosis: A Real‐World Perspective

**DOI:** 10.1111/ene.70409

**Published:** 2025-11-25

**Authors:** Pasquale Paletta, Paola Mosconi, Roberto Bergamaschi, Claudia Santucci, Vito Lepore, Eleonora Tavazzi, the Italian Multiple Sclerosis and Related Disorders Register Centers Group and the Scientific Committee of the Italian Multiple Sclerosis and Related Disorders Register

**Affiliations:** ^1^ Public Health Unit, Scientific Research Area Fondazione Italiana Sclerosi Multipla Genoa Italy; ^2^ Laboratory for Medical Research and Consumer Involvement Istituto di Ricerche Farmacologiche Mario Negri IRCCS Milan Italy; ^3^ Multiple Sclerosis Centre IRCCS Mondino Foundation Pavia Italy; ^4^ Department of Clinical Sciences and Community Health University of Milan Milan Italy

The growing visibility of transgender people, together with the parallel rise in scientific interest in transgender healthcare, underscores the need for standardized reporting and methodological approaches in both clinical practice and research.

Efforts have been made to harmonize the classification of treatments, diseases and conditions, but some features are no longer sufficient to refer to the complexity of real‐world settings. Despite the number of tools available [1], common guidelines regarding the clinical characterization of transgender populations in real‐world settings are still lacking. Information such as gender, sexual orientation or other variables that can potentially influence the disease course are rarely collected in addition to biological sex.

Since sex is related to multiple sclerosis (MS) etiology and gender can have an impact at a psychological, social and clinical level, they both need to be properly addressed. Collecting only sex at birth risks the loss of valuable information concerning clinical history, disease course and treatment of people with MS (pwMS) [2]. A transgender characterization is considered within the Italian Multiple Sclerosis and Related Disorders Register (RISM), a nation‐based real‐world project collecting more than 95,000 cases [3].

RISM collects personal and clinical data, and subjects are identified via their government‐assigned tax identifier that includes sex at birth. RISM uses the Medical Dictionary for Regulatory Activities (MedDRA) to report medical conditions/clinical events, and to characterize the transgender population (https://www.meddra.org/news‐and‐events/news/english‐meddra‐version‐280‐now‐available).

The label “Gender‐reassignment therapy” allowed us to identify a subject who is gender transitioning and two who completed the path by rectifying their personal data. The characterization of these cases is predominantly clinical (onset, diagnosis, pharmacological treatments, visits and relapses); no information has been found on non‐pharmacological treatments (e.g., psychotherapy) nor laboratory tests (e.g., hormones dosages). These three cases were identified in only one out of the 157 MS centers currently contributing to RISM. All of the items reported as “Lowest Level Term” of the MedDRA's branches organization are intuitive and easy to pick, while issues arise when it comes to the higher levels of the classification. In fact, many items are misleading and/or medicalizing (i.e., sexual identity disorders, psychiatric disorders) affecting the process of data collection.

Since MS is a female‐predominant disease, in which sex hormones have a relevant role, the health status of transgender pwMS is particularly relevant. In case of sex/gender transition hormonal and gender‐affirming therapies may induce/exacerbate the disease itself [2]. Furthermore, gender‐affirming hormone use may have a different impact based on the biological sex [4]. It is essential to collect these data for both personalized treatment and public health purposes. Indeed, although studies have been conducted on the experiences and perceptions of LGBTQIA+ pwMS [5], studies investigating their clinical aspects are scarce.

Having all three cases reported in a single center suggests that training of clinical staff on transgender issues is crucial not only for data collection, but also for providing optimal healthcare. More fine‐grained data collection of personal data is urgently needed to better address the complexity of human life, enriching the quality of healthcare and clinical research.

## Author Contributions

Pasquale Paletta: conceptualization, data curation, writing – original draft, writing – review and editing; Paola Mosconi: conceptualization, writing – review and editing; Roberto Bergamaschi: conceptualization, writing – review and editing; Claudia Santucci: writing – review and editing; Vito Lepore: conceptualization, data curation, writing – review and editing; Eleonora Tavazzi: conceptualization, writing – original draft, writing – review and editing.

## Ethics Statement

All the methods and procedures were performed in accordance with the guidelines and ethical standards and with the 1964 Helsinki Declaration and its later amendments. The RISM was reviewed and approved by the Ethics Committee of the coordinating centre (University of Bari, reference numbers 0,055,587 and 0,052,885), and by the local ethics committees of all participant centres. Each person enrolled signed a written informed consent. This analysis does not contain any individual person's information.

## Conflicts of Interest

R.B. has served on scientific advisory boards for Biogen, Merck‐Serono, Novartis, Sanofi‐Genzyme; received travel and congress expenses sustained by Biogen, Bristol Myers Squibb, Janssen, Novartis, Merck‐Serono, Roche, Sanofi‐Genzyme; received honoraria for speaking engagement from Biogen, Bristol Myers Squibb, Janssen, Merck‐Serono, Novartis, Sanofi‐Genzyme; received research support from Biogen, Merck‐Serono, Novartis, Sanofi‐Genzyme. E.T. received speaker honoraria from Biogen. The other authors declare no conflicts of interest.

## Data Availability

The data that support the findings of this study are available from the corresponding author upon reasonable request.
